# Characterization of cadmium-tolerant endophytic fungi isolated from soybean (*Glycine max*) and barley (*Hordeum vulgare*)

**DOI:** 10.1016/j.heliyon.2021.e08240

**Published:** 2021-10-22

**Authors:** Lyudmila Ignatova, Aida Kistaubayeva, Yelena Brazhnikova, Anel Omirbekova, Togzhan Mukasheva, Irina Savitskaya, Tatyana Karpenyuk, Alla Goncharova, Dilfuza Egamberdieva, Alexander Sokolov

**Affiliations:** a Faculty of Biology and Biotechnology, Al-Farabi Kazakh National University, Almaty, 050038, Kazakhstan; b Leibniz Centre for Agricultural Landscape Research (ZALF), Müncheberg, Germany; c Center of Physico-Chemical Methods of Research and Analysis, Al-Farabi Kazakh National University, Kazakhstan

**Keywords:** Endophytic fungi, Soybean, Barley, Heavy metals, Cadmium tolerance, Bioaccumulation capacity

## Abstract

Cadmium stress disrupts plant-microbial interactions and reduces plant growth and development. In plants, the tolerance to stress can be increased by inoculation with endophytic microorganisms. The aim of this study was to investigate the distribution of endophytic fungi in various plant organs of barley and soybean and evaluate their Cd removal ability.

Two hundred fifty-three fungal strains were isolated from various organs of barley (*Hordeum vulgare* cv Arna) and soybean (*Glycine max* cv Almaty). The colonization rate ranged from 13.6% to 57.3% and was significantly higher in the roots. Ten genera were identified: *Fusarium, Penicillium, Aspergillus, Metarhizium, Beauveria, Trichoderma, Rhodotorula, Cryptococcus, Aureobasidium* and *Metschnikowia*. Twenty-three fungal strains have a Cd tolerance index from 0.24 to 1.12. Five strains (*Beauveria bassiana* T7, *Beauveria bassiana* T15, *Rhodotorula mucilaginosa* MK1, *Rhodotorula mucilaginosa* RH2, *Metschnikowia pulcherrima* MP2) with the highest level of Cd tolerance have minimum inhibitory concentrations from 290 to 2400 μg/ml. These fungi were able to remove Cd up to 59%. The bioaccumulation capacity ranged from 2.3 to 11.9 mg/g.

Selected fungal strains could be considered as biological agents for their potential application in the bioremediation of contaminated sites.

## 1 Introduction

Endophytes are microorganisms that colonize internal plant tissues without causing apparent disease symptoms. Fungal endophytes play a crucial role in plant growth and development through enhancing nutrient acquisition, and protecting plants from biotic and abiotic stress (Rodriguez et al., 2009; Mukasheva et al., 2016; Vyas and Bansal, 2018). The study of distribution, ecology and benefits of fungal endophytes may open new perspectives in agriculture development and environmental protection.

One of the important concerns in agriculture is heavy metal (HM) soil contamination that causes some ecological and environmental problems. HM pollution of soils contributes to the deterioration of the physical, chemical and biological properties of soil, decreasing the growth and yield of agricultural crops (Etesami, 2018). Agricultural soils may contain various HMs that come into the soil with fertilizers, pesticides, and plant growth stimulants (Marković et al., 2010).

Cadmium (Cd) is one of the most toxic metals among HMs and widespread pollutants of the surface soil layer (Belimov et al., 2005; Hashem et al., 2019). In earlier reports, it has been shown that a lower concentration of Cd (0.5 mg kg^−1^ of soil) is considered as a beneficial mineral element for plant growth (Kabata-Pendias and Pendias 2001; Shadmani et al., 2020, 2021). However, higher concentration causes a broad range of biochemical and physiological dysfunctions such as reduction in photosynthesis, water and nutrient uptake that leads to the decrease of crop production. Agricultural crops grown in soil contaminated with Cd are characterized by disturbances of development: chlorosis, growth inhibition, browning of root tips, etc (Prasad, 2004; Yadav, 2010; Shadmani et al., 2020, 2021). Agricultural crops may accumulate various levels of Cd, e.g. cereals (0.013–0.22 mg kg^−1^) and grasses (0.07–0.27 mg kg^−1^) (Kabata-Pendias and Pendias 2001; Shadmani et al., 2020, 2021).

In order to grow agricultural crops without accumulation of HMs in plant tissues, it is necessary to improve plants resistance to HM stress. It is well known that some microorganisms enhance crop productivity and plant tolerance to HM stress. Now, it has been established that cells of different microorganisms can accumulate HMs in amounts much higher than they need for them as nutrition components. The accumulation of metal leads to its significant concentration in cells with respect to the medium. Microorganisms are resistant to almost all HMs (Ayangbenro and Babalola 2017; Etesami 2018).

Microbial mechanisms implicated in the remediation of HM toxicity include biosorption of HM ions, immobilizing them outside the cell, or depositing them on the cell wall, complexation, crystallization, and changing the valence of metal ions. Immobilization of HMs can occur due to a change in pH and redox potential of the medium, methylation, and dealkylation, the mobilization of phosphates or the production of polysaccharides, siderophores and other substances (Ayangbenro and Babalola 2017; Aryal 2020). Additionally, microorganisms promote the launching of antioxidant protective reactions in plants and the improvement of nutrient uptake, which is eventually manifested in increasing plant stress resistance and productivity (Shadmani et al., 2020).

A large number of studies are dedicated to the ability of using fungi to remove Cd from various polluted sites. The ability of active Cd removal is described for many typical representatives of soil and endophytic fungal microflora – *Aspergillus* (Chakraborty et al., 2014; Fazli et al., 2015: Manguilimotan and Bitacura, 2018; Alothman et al., 2019)*, Alternaria*, *Microdochium*, *Bipolaris*, *Alternaria*, *Pleosporales Fusarium Paecilomyces Clonostachys*, *Epicoccum* (Shadmani et al., 2020, 2021)*, Microsporum, Terichoderma* (Fazli et al., 2015), *Fomitopsis, Trichoderma, Rhizopus* (Oladipo et al., 2018), *Beauveria* (Fomina et al., 2005; Kameo et al., 2000), *Paraphaeos, Pyrenochaeta, Rhizopycnis* (An et al., 2015), *Penicillium* (Deng et al., 2017; Li et al., 2016; Manguilimotan and Bitacura, 2018; Alothman et al., 2019), *Candida* (Châu, 2013; Rehman et al., 2010), *Cryptococcus* (Châu, 2013), *Exophiala* (Zhao et al., 2015), *Rhodotorula* (Li and Yuan, 2008), *Zygosaccharomyces, Saccharomyces* (Li et al., 2014), *Aureobasidium* (Mowll and Gadd, 1984).

Soybean and barley are major world crops, however, the knowledge of their fungal, especially yeast, endophytic community is very limited. Considering the high toxicity of Cd and importance of endophytic fungi for plants grown in Cd contaminated soils, the aim of our work was to study the distribution of endophytic fungi in various plant organs of barley (*Hordeum vulgare* cv Arna) and soybean (*Glycine max* cv Almaty) and evaluate their Cd removal ability. Objectives: 1. To study the taxonomic composition and features of the distribution of endophytic fungi of soybean (*Glycine max* cv Almaty) and barley (*Hordeum vulgare* cv Arna) depending on the plant organs. 2. To determine Cd tolerance and minimum inhibitory concentration (MIC) of strains. 3. To investigate Cd removal ability and bioaccumulation of fungal strains. 4. To evaluate effect of Cd on morphological characteristics of fungi.

## 2 Materials and methods

### 2.1 Sampling site and sample collection

Two plant species Barley (*Hordeum vulgare* cv Arna) and Soybean (*Glycine max* cv Almaty) were selected for investigation and collected from the sampling site which is located in Turgen, Almaty region, Kazakhstan (43°27′N 77°34′E, 817 m a.s.l.). Ten healthy (without symptoms of disease) plants were collected randomly, taken in sterile bags to the laboratory, and processed within 24 h. Samples of chestnut soil were collected from agriculture area during the growing season. From each plot, litter and vegetation were removed from the soil surface then four to five soil cores (∼2.5 × 10 cm) were collected from the 2.5 × 2.5 m core subplots. The sub-samples were homogenized into a single sample. Samples were allowed to air dry in paper bags and then sieved (2 mm) to remove rocks, plant root fragments, and break up large clumps of soil. Then samples were transferred to laboratory for analyses.

### 2.2 Isolation of endophytic fungi

Endophytic fungi were isolated from plant tissues using the traditional fragment-plating technique (Vaz et al., 2014). Large fragments of healthy plant parts were first washed under running tap water for 30 min to remove soil residue and dust, and were surface-sterilized by dipping in 70% ethanol for 1 min and 3% NaCl solution for 2 min, followed by washing with sterile distilled water (2 min). Sterile plant parts were cut into small pieces (about 5 × 5 mm) with a sterile scalpel and were aseptically transferred to plates (10 fragments per plate) containing potato-dextrose agar (PDA) medium supplemented with 50 mg/L streptomycin and 50 mg/L tetracycline to avoid isolating endophytic bacteria. A total of 110 root, 110 leaf and 110 stem segments from each plant species were obtained. To test the effectiveness of the surface sterilization, 100 μl of the water used during the final rinse was plated on PDA and incubated at 25 °C. The plates were incubated in the dark at 25 °C for 21 days and checked daily. Any present fungi was isolated, purified and then maintained at 4 °C on PDA slopes for further studies.

The colonization rate (CR) was calculated as the total number of plant tissue segments colonized by one or more endophytic fungi divided by the total number of segments incubated, and expressed as percentage. The isolation rate (IR) used to represent a measure of fungal richness in a given sample was calculated as the number of isolates obtained from segments divided by the total number of segments. The distribution of taxa was expressed using the relative frequency (RF), which calculated as the number of isolates of one species divided by the total number of isolates, and expressed as percentage (Huang et al., 2008).

### 2.3 Identification of fungal endophytes

Morphological identification of endophytic filamentous fungi was based on the morphology of the culture colony, growth rate, characteristics of spores and reproductive structures (Ellis, 1976; Barnett and Hunter, 1998). The standard biochemical and morphological test procedures were used in yeast identification (Kurtzman et al., 2011).

### 2.4 Molecular identification of strains

Fungal cultures were grown in potato-dextrose agar (PDA) medium at 25 °C. DNA extraction from fungal tissues was carried out according to the procedure described by Wilson (2001). Cell lysis was achieved enzymatically by adding 567 μL TE buffer (10 mM Tris-HCl, 1 mM EDTA; pH 8.0), 30 μL 10% SDS and 3 μL proteinase K (20 mg mL−1) and incubated at 37 °C for 1 h. DNA was extracted with phenol/chloroform/isoamyl alcohol (ratio 25:24:1). The DNA was finally precipitated with (0.6:1 v/v) isopropanol and immediately centrifuged at 17.000 g for 10 min before air-drying and resuspending. After DNA extraction and purification its concentration was measured using NanoDrop spectrophotometer (Thermo Fisher Scientific, USA) at 260 nm and was normalized to 30 ng/μl.

The PCR primers were used: ITS4-5′-TCCTCCGCTTATTGATATGC-3′, ITS5-5′-GGAAGTAAAAGTCGTAACAAGG-3á (White, 1990), following previously described amplification conditions (Ignatova et al., 2015). Electrophoresis was carried out with 1% agarose gel in TAE. The amplifications products were purified by the enzymatic method and sequenced using BigDye®Terminator v3.1 Cycle Sequencing Kit (Applied Biosystems, USA) (Ignatova et al., 2015). The ITS sequences were analyzed in NCBI GenBank (https://www.ncbi.nlm.nih.gov) using BLAST. Sequences were aligned using ClustalW. Phylogenetic trees were constructed using the Neighbor-Joining method in Mega 6.0 software package (Tamura et al., 2013). Bootstrap analysis with 1000 replicates was carried out (Chernick and LaBudde, 2011).

### 2.5 Soil analysis

The soil pH was determined using an ionomer I-160M (“Antech”, Belarus). The soil sample was mixed with water at a ratio of 1:5 and the pH of the suspension was measured after 1 h of shaking.

Soil organic C and total N were determined using the dry combustion procedure described by Schepers et al. (1989).

To determine moisture content the soil samples were weighed (“wet weight”) and then were dried at 60 °C for 24 h. Next, the samples were weighed again (“dry weight”). Soil moisture was calculated using Eq. (1):

(1) $$\text{Moisture}\;\text{content}=\frac{\text{wet}\;\text{weight}\left(\text{g}\right)-\text{dry}\;\text{weight}\left(\text{g}\right)}{\text{dry}\;\text{weight}\left(\text{g}\right)}$$

The sieved soil samples were dried at 70°С for 24 h. One gram of soil was treated with 10 mL concentrated HNO_3_ heated up to dryness and then cooled. This procedure was repeated with another 10 mL concentrated HNO_3_ followed by 10 mL of 12 N HCl. Then the digested soil samples were warmed in 20 mL of 2 N HCl to redissolve the metal salts. Extracts were filtered using Whatman filter paper no. 40, and then the volume was adjusted to 25 mL with 1.5% HNO_3_. The concentration of HMs (Cd, Zn and Pb) in the digested soil samples was determined using atomic absorption spectrometer “Analyst 400” (PerkinElmer, Germany).

### 2.6 Determination of Cd tolerance and minimum inhibitory concentration

The tolerance of fungal isolates to Cd was determined by spot plate method. The endophytic fungal cultures were inoculated onto PDA medium agar plates in which the concentration of Cd was 100 μg/ml achieved by adding Cd(NO_3_)_2_. PDA medium agar plates without Cd were used as controls. Incubation was conducted at 25 °C for 14 days. Tolerance of fungi was studied by the determination of tolerance index (TI) and minimum inhibitory concentration (MIC). TI of the isolates was calculated as the diameter of test fungal colony on Cd incorporated medium divided by the diameter of the colony on medium without Cd. Fungal Cd tolerance was rated in the following way: 0.00–0.39 (very low tolerance), 0.40–0.59 (low tolerance), 0.60–0.79 (moderate tolerance), 0.80–0.99 (high tolerance) and 1.00 – >1.00 (very high tolerance) (Oladipo et al., 2018).

To determine MIC the different metal concentrations (from 100 to 2000 μg/ml) were used. The culture of test fungus (10^6^ CFU/ml) was inoculated on metal and control plates in triplicate. The MIC was defined as the lowest concentration at which no viable colony-forming units (CFU) were observed after 14 days incubation at 25 °C.

### 2.7 Cd bioaccumulation assay

To determine the Cd bioaccumulation by fungi, standard procedure as described by Pan et al. (2009) was adapted. The fungal cultures were inoculated into PD liquid medium with a concentration of Cd 100 μg/ml. The medium without adding Cd was used as a control. Flasks were shaken at 25 °C and 160 rpm for 7 days, the biomass was separated by the filtration (for filamentous fungi) or centrifugation (for yeasts), dried at 80 °C, and weighted.

The intracellular Cd bioaccumulation (I) and cell-surface Cd bioaccumulation (C) were determined by the fungal cells and the supernatant, respectively.

Localization of Cd (intracellular (I) and cell-surface (C)) was determined as described previously (Li et al., 2013). Briefly, the amount of Cd associated with the cell biomass was fractionated as the Ethylenediamine tetraacetic acid (EDTA) washable fraction, present at the cell surface, and the EDTA non-washable fraction, present intracellularly. The cells were resuspended for 30 min in 20 mmol/L EDTA solution for desorption of heavy metal from the cell surface and centrifuged. The amount of heavy metals bioaccumulated intracellularly (I) and on the cell surface (C) was determined by the digested fungal cells and the supernatant, respectively. The cells and supernatants were solved in 67% HNO_3_. The HNO_3_ solution was evaporated and the solids were redissolved in 0.1M HCl. The Cd concentration was measured by an atomic absorption spectrometer “Analyst 400” (PerkinElmer, Germany). The operating parameters were set as recommended by the manufacturer. Atomic absorption measurements were carried out in an air-acetylene flame. The following conditions were used: absorption line: Cd, 228.8 nm; slit widths, 0.5 nm; and lamp currents, 4 mA.

Cd removal rate (R) and total Cd bioaccumulation capacity (Q) were calculated by using following equations (Eq. (2), Eq. (3)):

(2) R = (1–C/C_0_)×100

(3) Q = (C_0_–C)V/W

where R is Cd removal rate in the (PDA) medium (%), Q is the bioaccumulation amount of mycelia (mg/g), C is the final concentration of Cd (mg/l), C_0_ is the initial concentration of Cd used (mg/l), V is the volume of the liquid medium (0.05 l in the experiments), and W is the fungal biomass (g).

### 2.8 Statistics

The data were processed by the standard methods of analysis of variance (ANOVA) using the software Statistica version 10.0 (StatSoft Inc., USA). Tukey's honestly significant difference (HSD) test (at p < 0.05) was performed to analyze statistical differences and to discriminate between means. All experiments were performed in triplicate. The differences between means were considered statistically significant if the p value was lower than 0.05.

## 3 Results

### 3.1 Isolation of endophytic fungi from *Glycine max* and *Hordeum vulgare*

To evaluate the distribution and diversity of fungal endophytes in crops, a total of 253 isolates were obtained from different plant organs. Among them 158 were isolated from *Glycine max* cv Almaty and 95 from *Hordeum vulgare* cv Arna. The colonization rate (CR) and isolation rate (IR) of endophytic fungi ranged from 13.6% to 57.3% and 0.18–0.75, respectively (Table 1). A perfect positive correlation between CR and IR was observed (r = +1.0, *p* = 0.00, n = 8). CR in the roots was significantly higher than in stems and leaves for both plant species. 135 endophytic fungi were isolated from the roots, 73 from stems and 45 from leaves (Table 1).

**Table 1:** Number, colonization rate (%) and isolation rate of endophytic fungi from different plant organs of soybean (*Glycine max* cv Almaty) and barley (*Hordeum vulgare* cv Arna).

	Soybean (*Glycine max* cv Almaty)				Barley (*Hordeum vulgare* cv Arna)			
	Root	Stem	Leaf	Total	Root	Stem	Leaf	Total
No. of segments plated	110	110	110	330	110	110	110	330
No. of segments colonized by endophytic fungi	63	39	23	125	41	17	15	73
No. of endophytic fungi isolated	82	51	25	158	53	22	20	95
CR (%)	57.3	35.5	20.9	37.9	37.3	15.5	13.6	22.1
IR	0.75	0.46	0.23	0.48	0.48	0.20	0.18	0.29

Based on the morphological characteristics, the isolated fungi can be divided into 10 genera. Among the identified taxa, 6 belonged to the filamentous fungi (*Fusarium, Penicillium, Aspergillus, Metarhizium, Beauveria.* and *Trichoderma*) and 4 to yeasts, among which, the genera *Rhodotorula* and *Cryptococcus* belong to basidiomycete affinity, and *Aureobasidium* and *Metschnikowia* are ascomycetic yeasts. Among all 10 taxa, six were found in both soybean and barley.

**Figure 1:**
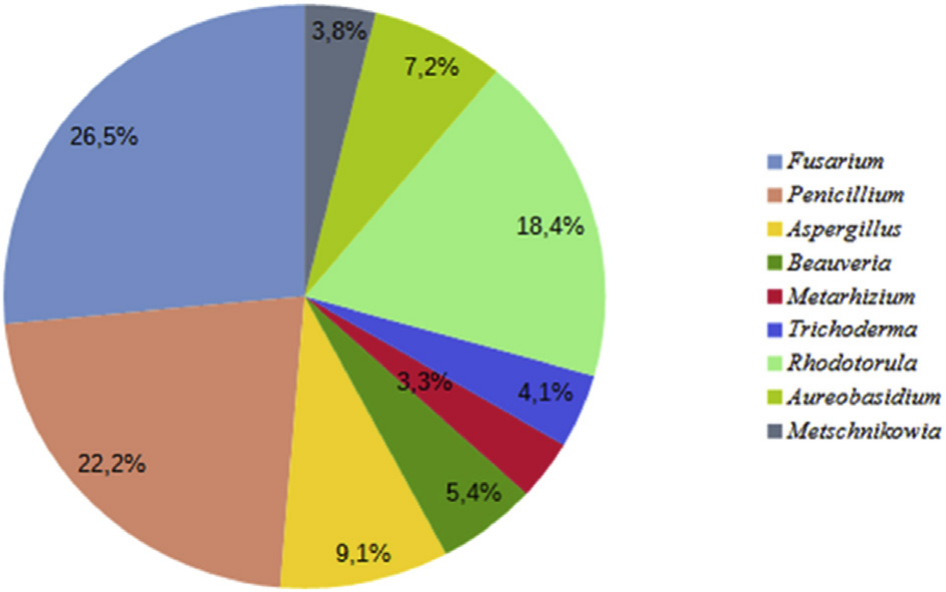
Relative frequency of different endophytic taxa isolated from soybean (*Glycine max* cv Almaty).

Among the endophytic fungi isolated from soybean, *Fusarium, Penicillium* and *Rhodotorula* were the most frequently isolated genera with RF of 26.5%, 22.2% and 18.4%, respectively. The least frequently found genera were *Trichoderma, Metarhizium* and *Metschnikowia* (Figure 1).

**Figure 2:**
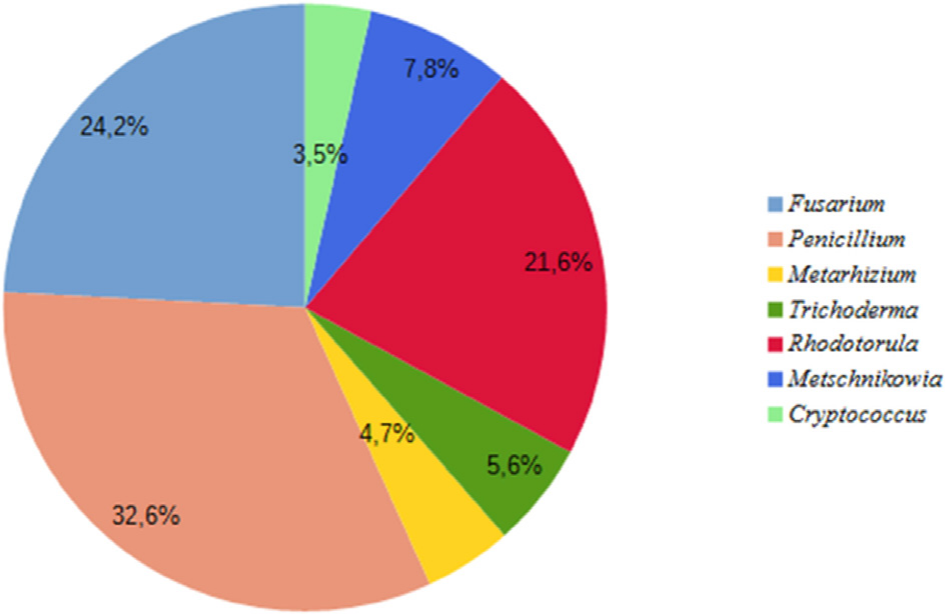
Relative frequency of different endophytic taxa isolated from barley (*Hordeum vulgare* cv Arna).

The endophyte community of barley was composed of 95 fungal isolates that belong to seven genera. *Penicillium* was the dominant endophytic genus with RF of 32.6%. Representatives of *Fusarium* and *Rhodotorula* genera also were the most frequently isolated fungi, constituting 24.2% and 21.6 % of the fungal communities of barley, respectively (Figure 2).

### 3.2 Soil HM content analysis

The total content of HMs was determined in the investigated samples of dark chestnut soil. As can be seen from Table 2, the excess of maximum permissible concentration (MPC) is detected only for Cd. The Cd content in studied soils was 2.5 and 3.92 times higher than MPC (Table 2).

**Table 2:** General physical and chemical parameters in soils under different crops.

Soil sample	Texture	Organic C (%)	Total N (%)	Moisture content	pH	Total content of HM (mg/kg)		
						Cd	Zn	Pb
Soil under soybean crops	Light loam	2.7 a	0.2a	0.062 a	8.4 ± 0.2 a	1.96 ± 0.05 c	152.51 ± 6.24 a	24.97 ± 1.12 a
Soil under barley crops	Light loam	2.8 a	0.2a	0.063 a	8.3 ± 0.1 a	1.25 ± 0.04 b	148.32 ± 7.35 a	22.17 ± 0.09 a
Maximum permissible concentration for HM						0.5 a	220.0 b	32.0 b

Data are presented as mean ± S.D. Different letters in the same column indicate statistically significant differences among values according to Tukey HSD test at the p < 0.05.

### 3.3 Cd tolerance and MIC of fungal endophytes

A total of 253 strains of endophytic fungi was isolated and screened from the initial (100 μg/ml) level of Cd supplemented media. Among them, 23 fungal strains showed tolerance to Cd. Significant differences in the Cd tolerance of fungi were revealed. TI ranged from 0.24 to 1.12. Of the fungi studied, one strain showed very high tolerance – *Beauveria bassiana* Т15 with TI - 1.12. High level of tolerance was observed for 4 strains: *Beauvera bassiana* T7 and *Metschnikowia pulcherrima* MP2 with TI = 0.90; *Rhodotorula mucilaginosa* МК1 and *Rhodotorula mucilaginosa* RH2 with TI = 0.88 (Table 3).

**Table 3:** Cadmium tolerance index of fungal endophytes under 100 μg/ml of Cd.

Genera	Strains	Tolerance index
*Fusarium* sp.	EF9	0.45
	EF15	0.51
	EF33	0.34
*Metarhizium* sp.	An1	0.41
*Beauveria* sp.	T7	0.90
	T15	1.12
*Trichoderma* sp.	ED7	0.54
	D11	0.48
*Penicillium* sp.	EF2	0.46
	EF3	0.37
	Pb11	0.52
	Pb14	0.24
*Aspergillus* sp.	AC4	0.49
	EF19	0.58
*Cryptococcus* sp.	LC3	0.64
*Rhodotorula* sp.	EY2	0.75
	EY3	0.79
	МК1	0.88
	RH2	0.88
*Aureobasidium* sp.	C7	0.73
	C10	0.64
*Metschnikowia* sp.	MP1	0.75
	MP2	0.90

Five fungal strains with the highest level of metal resistance were selected for further studies. Determination of the MICs demonstrated that the *Beauveria bassiana.* T15 strain exhibited maximum tolerance to Cd (2400 μg/ml). MICs were slightly lower for the *Beauveria bassiana* T7 (1400 μg/ml) and *Rhodotorula mucilaginosa* МК1 (1460 μg/ml) strains. For the *Rhodotorula mucilaginosa* RH2 and *Metsсhnicоwiа pulcherrima* MP2 strains, MICs were 430 μg/ml and 290 μg/ml, respectively (Table 4).

**Table 4:** Minimum inhibitory concentration of cadmium for fungal endophytes.

Strains	MIC (μg/ml)
*Beauveria bassiana* T7	1400
*Beauveria bassiana* T15	2400
*Rhodotorula mucilaginosa* МК1	1460
*Rhodotorula mucilaginosa* RH2	430
*Metsсhnicowiа pulcherrima* MP2	290

### 3.4 Cd bioaccumulation by active fungal endophytes

The fungi were able to remove up to 58.7% of Cd after 7 days incubation in liquid medium with the addition of 100 μg/ml Cd. The *Beauveria bassiana* T7 strain demonstrated the highest level of Cd removal ability compared to other strains. Cd removal rate (R) of yeast strains was a little lower (38.1–48.5%). The lowest R was detected for *Beauveria bassiana* T15 (7.4%) (Table 5).

**Table 5:** Cadmium removal ability and bioaccumulation of fungal strains.

Strains	Removal ability (R), %	Bioaccumulation capacity (Q), mg/g	Cell-surface bioaccumulation (C), %	Intracellular bioaccumulation (I), %
*Rhodotorula mucilaginosa* RH2	38 ± 2 b	8.9 ± 0.4 c	33 ± 2 b	67 ± 4 b
*Rhodotorula mucilaginosa* MK1	49 ± 3 c	11.9 ± 0.6 d	38 ± 3 b	62 ± 3 b
*Metsсhnicоwiа pulcherrima* MP2	39 ± 1 b	6.0 ± 0.3 b	19 ± 1 a	81 ± 5 c
*Beauveria bassiana* T15	7 ± 1 a	2.3 ± 0.1 a	79 ± 5 c	21 ± 2 a
*Beauveria bassiana* T7	59 ± 4 d	11.0 ± 0.5 d	22 ± 1 a	78 ± 5 c

Data are presented as mean ± S.D. Different letters in the same column indicate statistically significant differences among values according to Tukey HSD test at the p < 0.05.

Cd bioaccumulation capacity ranged from 2.3 ± 0.1 to 11.9 ± 0.6 mg/g depending on the fungal strain. The highest Q value was detected for *Rhodotorula mucilaginosa* MK1 and *Beauveria bassiana* T7 (Table 5).

**Figure 3:**
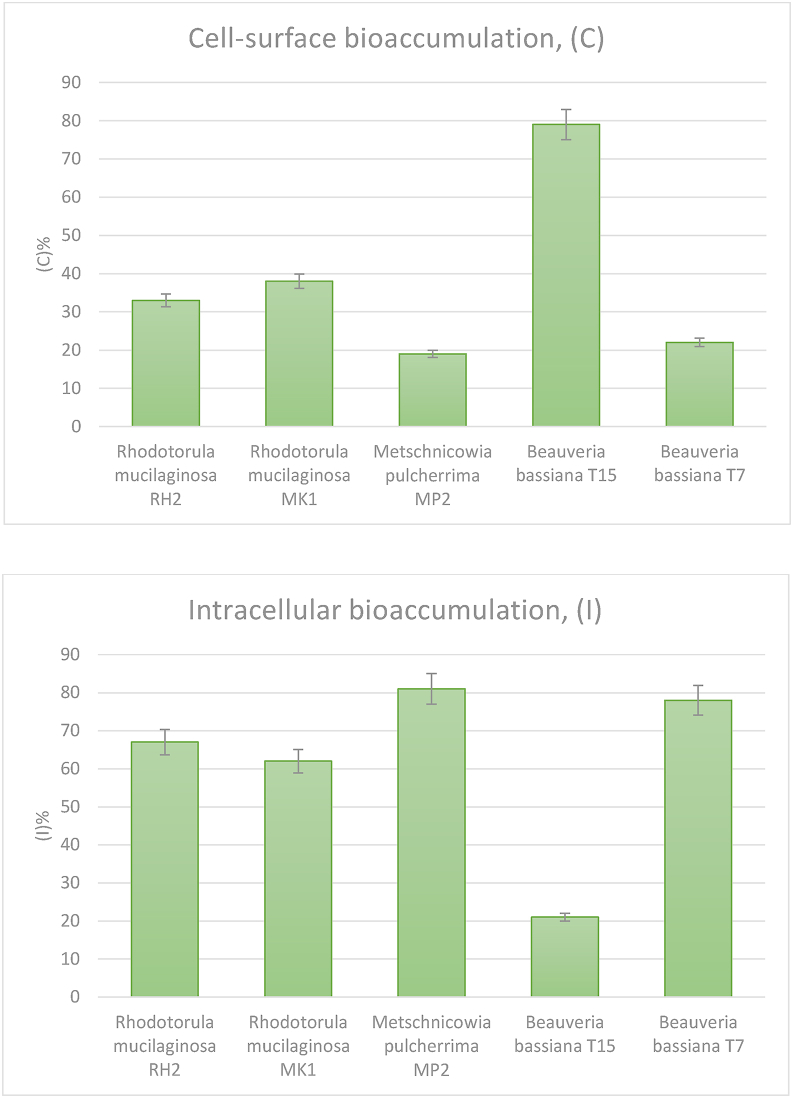
Cadmium cell surface and intracellular bioaccumulation of fungal strains.

Depending on the localization of accumulated Cd (intracellular and on the cell surface), it was shown that strains in a greater degree accumulate Cd intracellularly (I = 62–81%). The exception was the strain *Beauveria bassiana* T15, for which C = 79%, I = 21 % (Figure 3).

### 3.5 Effect of Cd on morphological characteristics of fungi

When growing on media amended with 100 μg/ml Cd, changes in the macro- and micromorphology of fungi were observed in comparison with the control.

**Figure 4:**
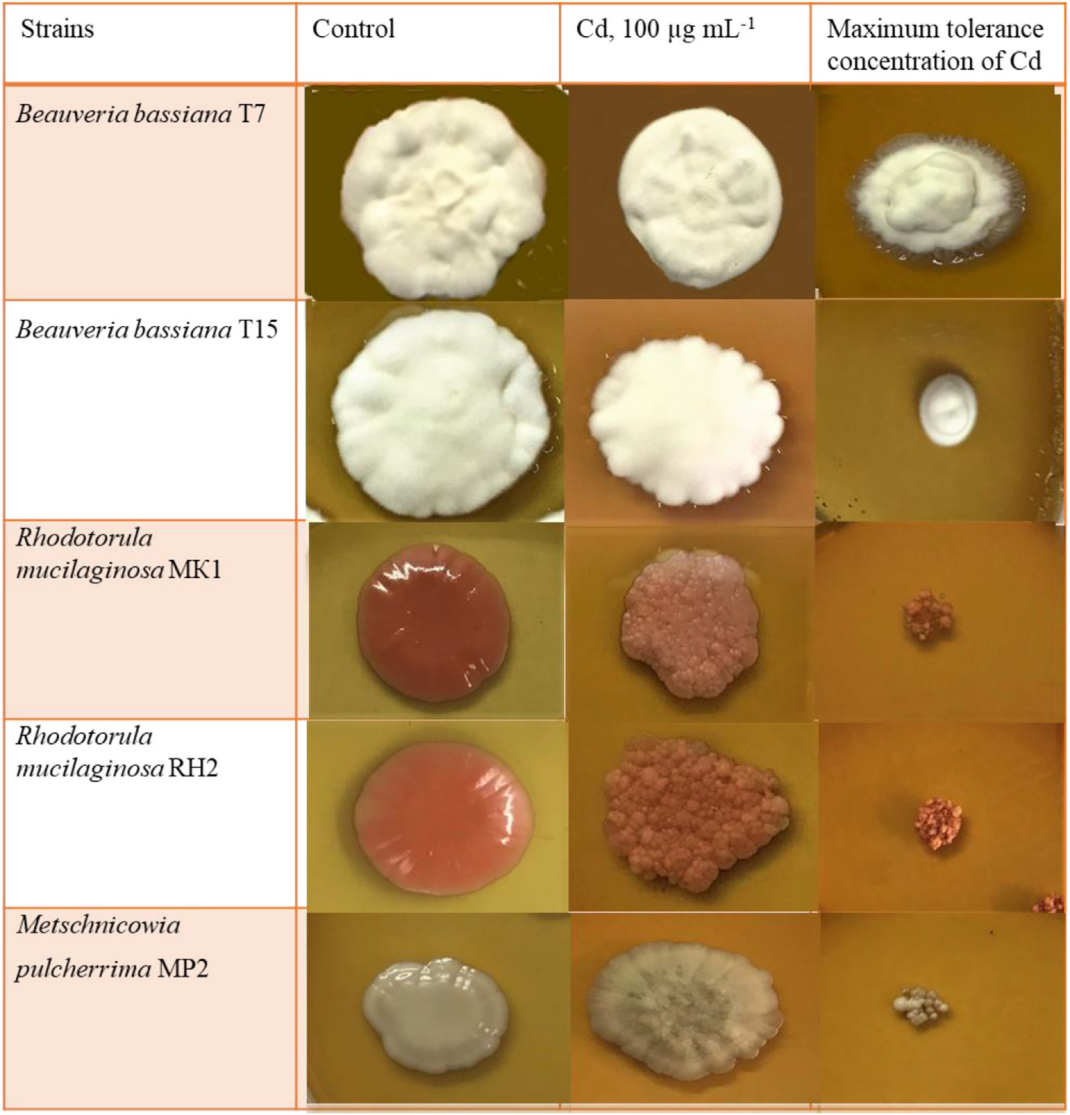
Colonies of fungal strains after 14 days of incubation on potato-dextrose agar (PDA) medium.

Colonies of filamentous fungi on medium with Cd became denser, with drops of exudate; had a pronounced substrate mycelium, growing into agar, which led to agar cracking (Figure 4). There was no change in color. The sporulating hyphae were less observed.

Colonies of yeast strains on Cd-incorporated medium became bumpy, pale, smaller, less shiny. Significant discoloration was observed (Figure 4). Yeast cells had dark intracellular inclusions, more distinct contour, became larger and more spherical compared to control.

**Figure 5:**
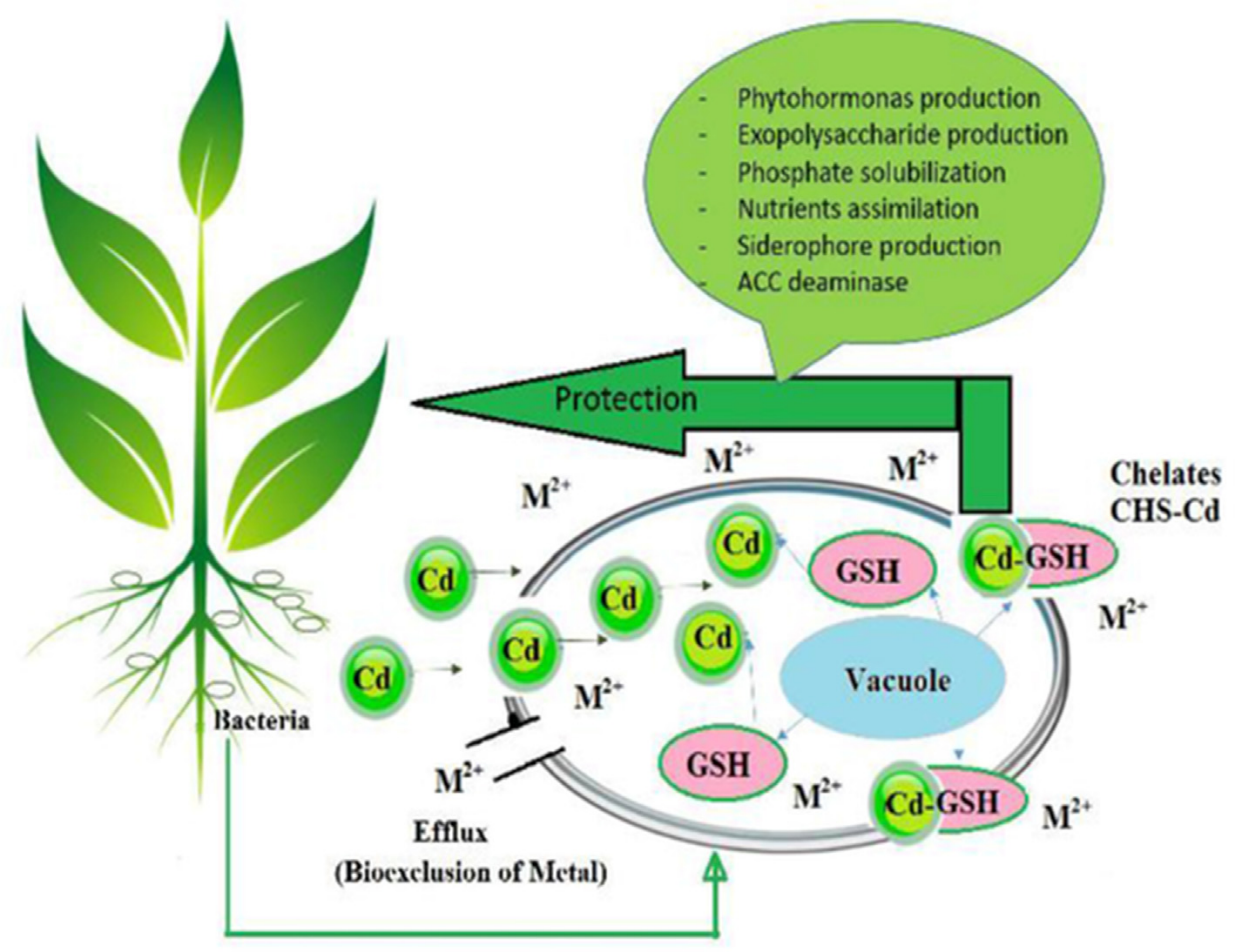
The mechanism of microbial-plant relationships under cadmium stress conditions.

Studies have shown that HM have a variety of effects on the composition of microbial cell components. Changes in the functioning of cells under stress caused by exposure to HM lead to the appearance of morphological abnormalities of MO, which is often expressed in a change in their shape and size. In most cases, these disorders are associated with dissociation of the processes of cell growth and division. The mechanism of interaction of micromycetes with Cd and the response of cells to stress can be presented as follows (Figure 5).

Up to 90% of the fungal cell wall consists of polysaccharides. The cell walls of micromycetes can be considered as a two-phase system consisting of a chitinous framework embedded in an amorphous polysaccharide matrix. The interaction of metal with the cell wall of fungi involves a complex mechanism that includes ion exchange, complexation, adsorption, and precipitation [(Tran and Popova, 2013)].

Polysaccharides and glycoproteins of the cell wall are involved in the binding of HM due to the functional groups. Cadmium is entered from the root system of plants to micromycetes cell via two pathways: biosorption and bioaccumulation. Mechanisms independent of metabolism are characteristic of both living and non-living cells, they include non-specific binding of metal to the cell surfaces of MO, mucus layers, extracellular matrices, etc. (passive absorption) and precipitation on the surface of the microbial cell (Chen et al., 2014). Mechanisms that depend on metabolism are typical only for living MO, they are based on the transfer of HM ions across the cell membrane, intracellular absorption and accumulation (bioaccumulation) (De Maria et al., 2011). Cations of HM can enter cells by diffusion through the cell wall. The penetration of HM ions into the cells of living organisms occurs by the mechanism of active transport, where Cd causes oxidative stress in the cell, mainly because it has a high affinity for thiols, especially for glutathione (GSH). HM binding to cells occurs by the mechanism of chelation. The GSH – Cd complex is transported to the vacuole via the Ycf1 protein, then the GSH complex is pumped out of the cell by Mrp1 protein. It inhibits Cd uptake by cells in the cytoplasm. Thus, GSH is processed to protect against HM and oxidative stress (Paula Daniela, 2009).

In addition to Cd chelation, micromycetes promote plant survival outside favorable environmental conditions by increasing the availability of essential elements such as iron, copper and zinc, increasing resistance to phytopathogens, stimulating phytohormone synthesis (Pishchik et al., 2015).

### 3.6 Molecular identification of fungal strains

Five selected fungal isolates, according their Cd tolerant ability, were identified by the ITS-region sequencing. The isolates were identified as *Beauveria bassiana* T7 (GenBank accession no. MG966197), *Beauveria bassiana* T15 (GenBank accession no. MG970260)*, Rhodotorula mucilaginosa* МК1 (GenBank accession no. MG966282), *Rhodotorula mucilaginosa* RH2 (GenBank accession no. MG969795), *Metschnikowia pulcherrima* MP2 (GenBank accession no. MG966451).

These strains of endophytic fungi were deposited to “Republican Collection of Microorganisms” CS MES RK (Nur-Sultan, Kazakhstan) and “Russian Collection of Agricultural Microorganisms” (RCAM, St.-Petersburg, Russian Federation).

## 4 Discussion

Studies of fungal endophytes in many environments are an active area for research. However, little is known about Cd-tolerant endophytic fungi and yeasts from soybean and barley. This work expands our understanding of the distribution features of endophytic fungi in soybean and barley, their Cd removal ability and the effect of Cd on morphological characteristics of these fungi.

Studies on the diversity and benefits of fungal endophytes display their huge impact on plant growth and development. HMs are the most widespread pollutants of the environment, which penetrate living cells, violate their vital functions and inhibit plant growth (Rodriguez et al., 2009). HM contamination of agricultural soils is typical not only for Kazakhstan, but also for many other countries (Tóth et al., 2016). We studied the soil samples under soybean and barley crops and found that the Cd content was 1.96 and 1.25 mg/kg, respectively, which exceeded the normal levels of Cd (Online Zakon Kz, 2004). High levels of toxic metal found in the studied agricultural soil samples are possibly associated with the introduction of various fertilizers, herbicides and pesticides containing HMs and other pollutants. High accumulation of Cd in crops leads to an increase in the Cd entrance to the food chain. HMs are not naturally decomposed, so the use of fungi with the metal removal ability is one of the possible solutions to this problem.

Soybean and barley plants were collected in agro-industrial firm “Turgen” which is located in Turgen, Almaty region, Kazakhstan. This firm is the leader in the production of agricultural crops in this region. Studies of the distribution of endophytic fungi in various plant organs of crops and evaluation of their Cd removal ability have never been conducted in this region.

In this study, the fungal colonization and diversity was influenced by the plant species. We found more endophytes from soybean plants. A total of 158 endophytic fungi from *Glycine max* cv Almaty and 95 isolates from *Hordeum vulgare* cv Arna were obtained. The possible reason for this result is related to soybean plant features. Thus, soybean is known as a rich source of beneficial endophytic fungi improving its fitness to survive under stress, including Cd contamination (Hamayun et al., 2017).

In the present study, the number of isolates from aboveground organs was less comparable to underground ones. Similar results were obtained from studies on other grassy plant species (Ghimire et al., 2011; Wearn et al., 2012; Jin et al., 2013), including barley (Hammami et al., 2016). Such differences in the distribution of endophytes in parts of plants can be explained by several reasons. Soil-borne fungi are typically much more diversified and prevalent than air-borne ones (Ghimire et al., 2011), which presumably may contribute to the fact, that belowground plant parts are more frequently colonized by endophytes. The other reason is that the main sources of easily accessible substrate are roots, so roots might be considered as a relatively stable environment adequate for many fungal species. Additionally, adverse factors such as desiccation, UV radiation and lack of nutrients affect aboveground plant organs, which could account for the more frequently colonization of roots comparatively to leaves and stems (Lindow and Brandl, 2003).

The isolation of endophytes from the different internal plant tissues showed the presence of representatives of 9 genera in *Glycine max* cv Almaty and 7 in *Hordeum vulgare* cv Arna. In the present study, the most frequently isolated genera were *Fusarium, Rhodotorula* and *Penicillium.* These fungi were isolated from both investigated crops and showed frequencies greater than 18.4%. Other genera (*Metarhizium*, *Beauveria, Trichoderma, Aureobasidium*, *Metschnikowia* and *Cryptococcus*) rarely occurred or only once either in soybean or in barley.

Previous studies showed that genera such as *Fusarium, Penicillium, Trichoderma, Aspergillus* and *Rhodotorula* revealed in this study also occur in other cultivated soybean plants (Impullitti and Malvick, 2013; Pimentel et al., 2006; Russo et al., 2016; Fernandes et al., 2015; de Souza Leite et al., 2013). The fungal endophytic communities of barley were represented by such genera as *Fusarium, Penicillium, Metarhizium, Trichoderma, Rhodotorula, Metschnikowia,* and *Cryptococcus,* which is consistent with previous studies of other authors (Murphy et al., 2015, 2018; Hammami et al., 2016). Species of the genera *Beauveria, Aureobasidium, Metschnikowia* and *Cryptococcus* as endophytic fungi were not found in soybean and barley in previous studies and were isolated from these plants for the first time.

According to the results, six strains were characterized by moderate metal tolerance: *Cryptococcus sp.* LC3, *Rhodotorula sp.* EY2, *Rhodotorula sp.* EY3, *Aureobasidium sp.* C10, *Aureobasidium sp.* C7, and *Metsсhnicоwiа sp.* MP1. Nine fungal isolates corresponded to the level of low tolerance, and three strains showed very low tolerance to Cd for which the value of TI did not exceed 0.37. After the first screening, 5 strains with the highest levels of Cd tolerance were selected for the next stage of the study: *Beauveria bassiana* Т7, *Beauveria bassiana* Т15, *Rhodotorula mucilaginosa* МК1, *Rhodotorula mucilaginosa* RH2, and *Metsсhnicоwiа pulcherrima* MP2. These strains have MICs from 290 to 2400 μg/ml, that similarly or higher than MICs for soil and endophytic fungal strains in other studies (Fazli et al., 2015; Châu, 2013; Deng et al., 2017; Chakraborty et al., 2014; An et al., 2015; Xu et al., 2015; Li et al., 2016; Rehman et al., 2010; Shadmani et al., 2021).

The most effective strains with higher accumulation ability were *Beauveria bassiana* T7 and *Rhodotorula mucilaginosa* MK1, with R-values 59 and 49%, respectively. In terms of bioaccumulation capacity, the strains *Rhodotorula mucilaginosa* MK1 and *Beauveria bassiana* T7 demonstrated high activity with the values of Q 11.9 and 11.0 mg/g. Obtained values are similar or higher than the results reported by other researchers (Fazli et al., 2015; Doku and Belford, 2015; Machido et al., 2016; Manguilimotan and Bitacura, 2018). The capacity of fungal isolates to accumulate Cd varied according to the fungal strain. The difference in these values can be related to the size and features of the cell surface of various fungi, as well as the initial concentration of Cd in the medium.

Fungal biomass interacts with metal, either through: (1) biosorption of metals into biomass (cell walls, pigments and extracellular polysaccharides) or (2) intracellular accumulation and sequestration; or (3) deposition of metal compounds on and/or around hyphae (Ayangbenro and Babalola 2017; Aryal 2020). In the present study, a greater amount of metal was accumulated intracellularly at Cd concentration of 100 μg/ml. Cd assimilation often takes place in a two-phase mode, when there is an initial non-volatile biosorption on the cell surface, followed by a slower energy-dependent intracellular accumulation (Li and Yuan, 2008; Li et al., 2013; Mowll and Gadd, 1984). It was shown that the accumulation of intracellular Cd increases with the concentration of metal in the medium to a certain extent (Li et al., 2014).

The action of the metal can affect the morphology and physiology of cells in various ways. In our studies, when cultivating yeasts on solid media containing Cd at a concentration close to MIC, a long delay in growth and changes in the appearance of the colonies occurred. Colonies became smaller, matte and paler, lost their luster. In addition, the changes concerned the micromorphology of yeasts. In the presence of Cd, an increase in the size of yeast cells, a thickening of the cell wall, and the presence of dark inclusions within the cells were observed. Similar results were shown in other studies for different yeast species (Li and Yuan, 2008; Rehman et al., 2010). In the case of filamentous fungi, mycelial compaction was established, the substrate mycelium became leathery, growing into agar, causing cracking of the nutrient medium. Morphological changes in the growth segment of hyphae and changes in the color of fungal cultures were noted by a number of authors (Baldrian, 2003; Fazli et al., 2015). When cultivating microorganisms on media in the presence of high concentrations HMs, an increase in cell size is most often noted, similar to the effect of other unfavourable chemical and physical factors on microorganisms (Fomina and Gadd, 2002). In addition, the effect of Cd on fungi results in lipid peroxidation, enzyme inactivation and membrane damage, eventually results in an unbalanced cellular redox status (Zhao et al., 2015).

## 5 Conclusion

The results showed that the distribution and diversity of fungi associated with soybean and barley depend on plant type. From 253 endophytic microorganisms isolated from plants, 5 strains (*Beauveria bassiana* T7, *Beauveria bassiana* T15*, Rhodotorula mucilaginosa* МК1, *Rhodotorula mucilaginosa* RH2, *Metschnikowia pulcherrima* MP2) with the highest levels of Cd tolerance and removal ability were selected. These isolates act as strains promising for alleviation of Cd stress and decrease the accumulation of metals in plant tissues.

## Declarations

### Author contribution statement

Lyudmila Ignatova; Yelena Brazhnikova; Dilfuza Egamberdieva: Conceived and designed the experiments; Wrote the paper.

Aida Kistaubayeva: Performed the experiments; Wrote the paper.

Anel Omirbekova; Togzhan Mukasheva; Irina Savitskaya: Contributed reagents, materials, analysis tools or data.

Tatyana Karpenyuk; Alla Goncharova: Performed the experiments; Analyzed and interpreted the data.

Alexander Sokolov: Performed the experiments; Contributed reagents, materials, analysis tools or data.

### Funding statement

This work was supported by the 10.13039/501100004561Ministry of Education and Science of the Republic of Kazakhstan: AP09261262 Biotechnology of microbial composition's creating to stimulate growth and increase the adaptive potential of agricultural plants.

### Data availability statement

No data was used for the research described in the article.

### Declaration of interests statement

The authors declare no conflict of interest.

### Additional information

No additional information is available for this paper.
